# Directionally hiding objects and creating illusions above a carpet-like device by reflection holography

**DOI:** 10.1038/srep08581

**Published:** 2015-02-26

**Authors:** Qiluan Cheng, Kedi Wu, Yile Shi, Hui Wang, Guo Ping Wang

**Affiliations:** 1 School of Physics and Technology, Wuhan University, Wuhan 430072, Hubei, P. R. China; 2 College of Electronic Science and Technology, Shenzhen University, Shenzhen 518060, Guangdong, P. R. China; 3 Institute of Information Optics, Zhejiang Normal University, Jinhua 321000, Zhejiang, P. R. China

## Abstract

Realization of a perfect invisibility cloak still challenges the current fabricating technologies. Most experiments, if not all, are hence focused on carpet cloaks because of their relatively low requirements to material properties. Nevertheless, present invisibility carpets are used to hide beneath objects. Here, we report a carpet-like device to directionally conceal objects and further to create illusions above it. The device is fabricated through recording a reflection hologram of objects and is used to produce a time-reversed signal to compensate for the information of the objects and further to create light field of another object so as to realize both functions of hiding the objects and creating illusions, respectively. The carpet-like device can work for macroscopic objects at visible wavelength as the distance between objects and device is at decimeter scale. Our carpet-like device to realizing invisibility and creating illusions may provide a robust way for crucial applications of magic camouflaging and anti-detection etc.

In terms of transformation optics^1^,^2^ and conformal mapping^3^, different kinds of invisibility cloaks have been proposed, designed and demonstrated theoretically and experimentally. To cloak objects, a shell configuration^4^,^5^,^6^,^7^,^8^,^9^,^10^ is designed to control electromagnetic waves to propagate around the enveloped objects while maintain original propagation wave front like passing through an equivalent free space. To overcome the limitation that objects to be hidden are generally enveloped by a cloak shell, complementary media -based distant cloaking devices are proposed and numerically demonstrated to hide an object outside the cloak shell^11^ and furthermore to create an optical illusion of changing one object into another object^12^,^13^. However, perfect invisibility cloaks require the constitutive materials with extreme properties such as anisotropy, inhomogeneity, singular dielectric parameters, or even negative index of refraction, which challenge the current fabricating technologies^14^,^15^, specifically in optical frequencies. So far, apart from a concept proof of metamaterial-based two-dimensional (2D) invisibility cloaks for hiding objects from millimeter-scale cylinder in the microwave region^4^ to living creatures with incoherent natural light^8^, as well as some testing experiments for creating illusions in microwave region^13^,^16^ and thermal heat^17^, most experiments, if not all, were focused on carpet cloaks-- a simplified model of shell cloaks, which can reshape the reflection light from a curve surface of the cloaks to make it looks like reflected from a plane mirror. As a result, when an object is placed beneath the curve surface of the carpet cloaks, it is unperceivable to the outside through reflection light^18^. Due to their relatively low requirements to material properties, different configurations of the carpet cloaks with 2D^19^,^20^,^21^ and three-dimensional (3D)^22^,^23^ structures by using manmade metal-contained metamaterials^19^ or metal-free all-dielectrics^20^,^21^,^22^,^23^ and even naturally available anisotropic materials^24^,^25^ are constructed with working frequency ranging from microwave frequencies^19^ to Terahertz, near-infrared and even visible^23^,^24^,^25^. The hidden objects also vary in size from wavelength scale^19^,^20^,^21^,^22^,^23^ to macroscopic^24^,^25^. Nevertheless, all of the carpet cloaks are used to hide objects beneath the carpets (Fig. 1a).

Here, we experimentally demonstrate a kind of carpet-like devices to directionally hide an object above them (Fig. 1b) and further to create an illusion of transferring the above object to another one (Fig. 1c)^26^. The carpet-like devices are reflection holograms made of commercially available all-dielectric materials. By producing a time-reversed signal to compensate for information of an object and further an additional light field of another object, the carpet-like devices can realize functions of hiding the object and creating illusion in the visible as the object is at centimeter scale in size and at a distance of several tens of centimeters away from the carpet-like devices. We believe these carpet-like devices to realizing invisibility and illusions should be more applicable in the fields such as magic camouflaging and anti-detection etc.

## Results

### Theoretical basis and fabricating procedures of carpet-like devices

So far, time-reversal principle has been widely exploited to produce phase-conjugation signals for not only imaging beyond the diffraction limit^27^,^28^, focusing light into a scattering medium^29^,^30^,^31^, but also concealing objects and creating illusions^32^,^33^. Experimentally, the time-reversed light beam can be created through either linear^29^,^30^,^31^,^32^,^33^ or nonlinear optical effects^34^ such as holography and four-wave mixing. However, all the above applications are generally based upon transmission detection. Here, we employ reflection holography to produce reflection time-reversal light to realize hiding objects and creating illusions above a carpet-like device through reflection observation.

**Figure 2:**
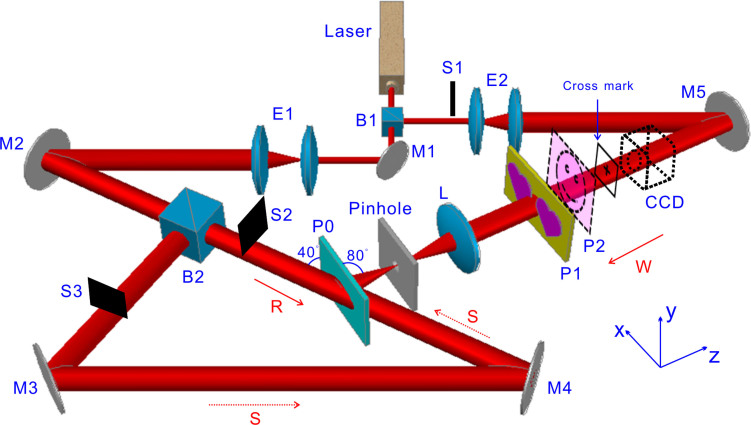
Experimental setup for constructing and characterizing the carpet-like devices. A light beam with wavelength λ = 632.8 nm from a He-Ne laser is divided into three parts: object light W, reference light R and reconstruction light S by beam splitters B1 and B2. M1-M5 are mirrors, E1 and E2 are beam expanders, S1, S2 and S3 are the shuttles, L is a lens with focal length f = 10 cm, P0, P1 and P2 are planes for recording plate, objects O_1_ and O_2_, respectively. The distance between object plane P1 and L is 20 cm, which equals to the distance between L and P0. So P0 is the one-to-one image plane of P1. The pinhole is used to block the stray light. A charge-coupled device (CCD) placed 20 cm away behind O_1_ is used to take images of objects and monitor the characterization of carpet-like devices. A cross mark is placed between the CCD and object plane P1 as a reference mark for monitoring the effect of the carpet-like devices on other objects nearby O_1_. The x-y plane of coordinate system is parallel to P1 and the z axis direction is anti-parallel to the light beam W. In exposure process, shuttles S1 and S2 are opened but S3 is closed, while in the process of characterizing the effects of finished carpet-like devices, shuttles S1 and S2 are closed but S3 is opened.

To construct a carpet-like device (C-1) for concealing object O_1_, we place O_1_ in plane P1 (Fig. 2) to record a reflection hologram of O_1_ in plane P0. While to get another carpet-like device (C-2) for creating optical illusions of changing object O_1_ to another object O_2_, we place objects O_1_ in plane P1 and O_2_ in plane P2 simultaneously in the recording procedure.

**Figure 3:**
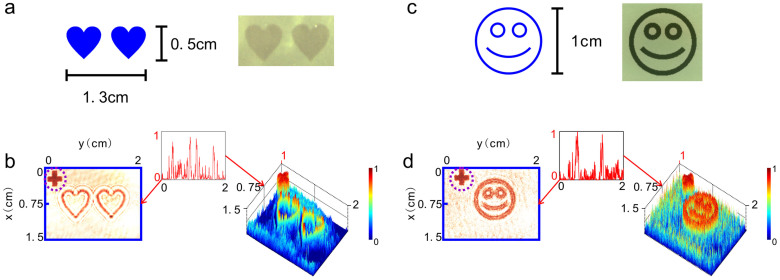
Sketches of object O_1_ and object O_2_ used in the experiments. (a) Sketch of object O_1_ (left) and its actual photograph (right): a two-heart picture with area 1.3 cm × 0.5 cm. (b) Planar image of object O_1_ (left hand panel) received by CCD and its 3D intensity profile (right hand panel) when O_1_ is placed in P1 plane of the optical setup and is directly illuminated by a plane light. (c) Sketch of object O_2_ (left) and its actual photograph (right): a smiling-face logo with diameter 1 cm. (d) Planar image of object O_2_ (left hand panel) received by CCD and its 3D intensity profile (right hand panel) when O_2_ is placed in P2 plane of the optical setup and is directly illuminated by a plane light. Insets of each picture are the line graphs of the corresponding images along the *y* axis at *x* = 0.75 cm.

Experimentally, object O_1_ is a transparent two-heart picture embossed on a glass substrate, while object O_2_ is an amplitude-modulated smiling face logo (see Methods). Figures 3a and 3c show the schemes and actual photographs of two objects O_1_ and O_2_, and the left hand panels of Figs. 3b and 3d present the images of O_1_ and O_2_, respectively, as they are directly illuminated by a laser beam at 632.8 nm. To quantitatively know the contrast of the images, we show in the right hand panels of Figs. 3b and 3d their 3D profiles and the insets intensity line graphs of the images along the *y* axis at *x* = 0.75 cm. We can see that in intensity line graph of O_1_, there are four feature peaks that correspond to the edges of two hearts, while in that of O_2_, two feature peaks correspond to the edges of smiling face. The dashed-line circled cross at the top left corner of the images is the image of a cross mark placed between object O_1_ and a charge coupled device (CCD) for reference.

After exposure, development, bleaching and drying, the carpet-like devices are finished (see Supplementary Table 2). Since the bleaching processing turns the composites of the recording medium into a dielectric material, the carpet-like devices obtained in our experiments are completely transparent.

### Characterization of C-1 in hiding object O_1_

**Figure 4:**
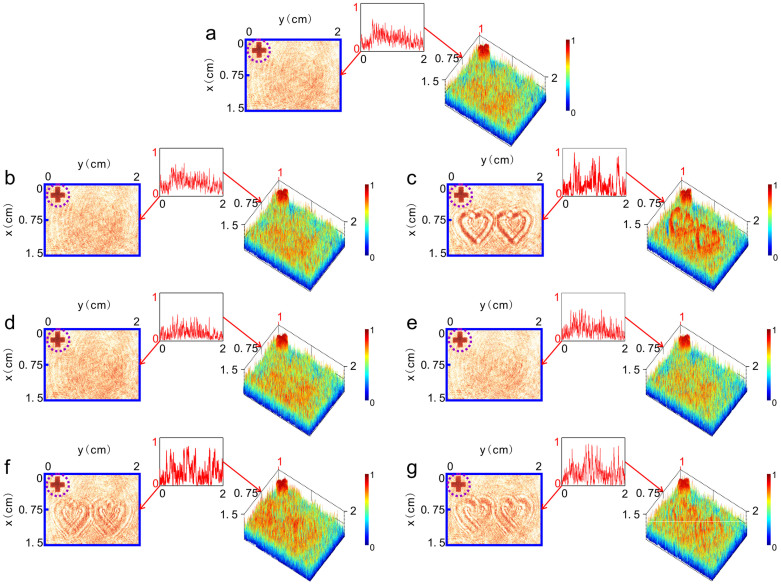
Experimental results of hiding object O_1_. (a) Planar image (left hand panel) received by CCD and its 3D intensity profile (right hand panel) when C-1 is precisely replaced in P0 plane of the optical setup. (b–c) Planar images (left hand panels) received by CCD and their corresponding 3D intensity profiles (right hand panels) as C-1 is deviated transversally 10 μm and 650 μm away from its original position along the x axis, respectively. (d–e) Planar images (left hand panels) received by CCD and their corresponding 3D intensity profiles (right hand panels) as C-1 is deviated longitudinally about ± 1 mm (approaching to or going away from the object) away from its original plane along the z axis, respectively. (f–g) Planar images (left hand panels) received by CCD and their corresponding 3D intensity profiles (right hand panels) when orientation deviation of C-1 is as small as 0.1° away from its original orientation around, respectively, the y axis in the P0 plane (Fig. 4f) or the x axis in the P0 plane (Fig. 4g). Insets in each figures are the line graphs of the corresponding images along the *y* axis at *x* = 0.75 cm. Dashed-line circled cross at the top left corner of each picture is the image of the cross mark placed between object O_1_ and CCD.

To examine the effect of C-1 in hiding object O_1_, we replace C-1 in plane P0 and maintain the position of O_1_ in plane P1. Fig. 4a shows the planar image received by CCD when C-1 is illuminated by reconstruction light S (left hand panel). From the figure we can hardly see any information of O_1_ except the background noise (caused by interference of parasitical light beams in the recording process). To get a quantitative description, we also show in the right hand panel the 3D intensity profile of the image and in the inset its line graph along the *y* axis at *x* = 0.75 cm. We find that, unlike the inset of Fig. 3b, there are no four feature peaks of O_1_ in the line graph except random back ground noise. It means that the time-reversed signal produced by C-1 indeed compensates for the scattering effect of O_1_ and makes it invisible. A movie illustrating the process of concealing object O_1_ by using C-1 is displayed in Supplementary movie 1.

In holographic conjugation technology, deviations of the replaced hologram from its original position and orientation etc. in the recording process may seriously destroy the function of the technology. We then check the effect of position and orientation deviations of carpet-like device C-1 on hiding object O_1_ above. The left hand panel of Fig. 4b shows the received planar image of CCD as C-1 is deviated transversally 10 μm away from its original position along the x axis. The right hand panel and inset are the 3D intensity profile of the image and its line graph along the y axis at *x* = 0.75 cm, respectively. We can see that there still exists no any information of O_1_. When the transversal deviation becomes as large as 650 μm away from its original position in the x axis, however, the profile of two hearts turns to observable (Fig. 4c, left hand panel) and the feature peaks of O_1_ begins to become to readable (Fig. 4c, inset).

Figures 4d and 4e exhibit the received planar images of CCD (left hand panels) and their corresponding intensity profiles (right hand panels) and line graphs along the y axis at *x* = 0.75 cm (insets) as C-1 deviates longitudinally about ± 1mm (approaching to or going away from the object) away from its original plane along the z axis, respectively. We find that such large longitudinal deviation is still tolerable for hiding object O_1_: it is still hard to discriminate the feature peaks of object O_1_.

For the effect of orientation deviation of C-1 on hiding object O_1_, we find that a little deviation from its original orientation seriously destroys the result. Our experimental results show that when the orientation deviation of C-1 is as small as 0.1° away from its original orientation around, respectively, the y axis in the P0 plane (Fig. 4f) or the x axis in the P0 plane (Fig. 4g), O_1_becomes visible, indicating that a small rotation deviation from its original orientation destroys the concealing effect of C-1. This is because, when the holographic plate is rotated by 0.1° along the x (y) axis, the diffraction light beam will move along the y(x) axis of about 0.7 mm on the plane of lens L, which is much larger than the tolerant deviation of the holographic plate to the transverse displacement deviation along the x(y) axis (about 650 μm).

It should be pointed out that, as mentioned before, any deviation of C-1from its original location may ruin its function of phase-conjugation signals. However, from above results we see that, in a certain range of displacement deviations occurring in the transversal and longitudinal directions, our carpet-like device still works well in hiding object. This can be attributed to the background noise of image in reconstruction.

Note that, although object O_1_ is concealed by C-1, from Figs. 4a–4g we see that, no matter object O_1_ is concealed or not by C-1, the cross mark placed between object O_1_ and CCD is always visible (dashed-line circled cross at the top left corner of each picture). This indicates that C-1 just plays the role of concealing the object to be hidden but has no effect on other objects nearby.

### Characterization of C-2 in transferring object O_1_ into O_2_

**Figure 5:**
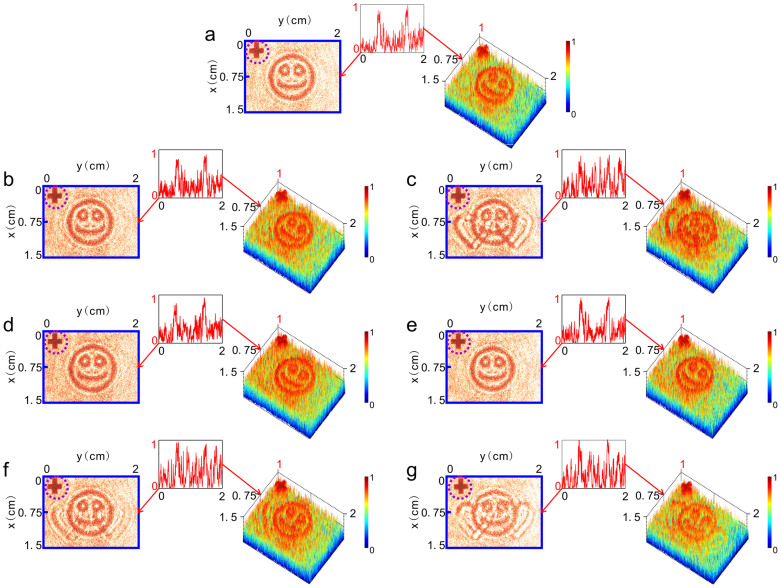
Experimental results of creating an illusion of transferring O_1_ into O_2_. (a–g) the same as in Figs. 4a–4g, respectively, for checking the effect of position and orientation deviations of carpet-like device C-2 on creating optical illusions of transferring O_1_ to O_2_.

Figure 5a presents the planar image (left hand panel) taken by CCD when only object O_1_ is fixed in P1 plane (object O_2_ is removed) but C-2 is replaced in P0 plane and is illuminated by reconstruction light S. We see that, although we only place object O_1_ in plane P1, what is received by CCD camera is not the image of object O_1_ but instead an image of object O_2_ (smiling-face picture). From its 3D profile (right hand panel, Fig. 5a) and line graph of the image along the *y* axis at *x* = 0.75 cm (inset, Fig. 5a), we see that not only there appear no the four feature peaks of object O_1_ (inset of Fig. 3b), but two feature peaks of O_2_ appear instead (inset of Fig. 3d). Consequently, we see that the carpet-like device C-2 can not only produce a time-reversed signal for compensating for the scattered light fields of O_1_ but further create an additional light field of O_2_ so that O_1_ is looked as another different object, O_2_. A movie illustrating the process of creating an illusion of transferring O_1_ to O_2_ by using C-2 is displayed in Supplementary movie 2.

Figures 5b–5g exhibit the same as in Figs. 4b–4g, respectively, for checking the effect of position and orientation deviations of carpet-like device C-2 on creating optical illusions of transferring O_1_ to O_2_. Similar to concealing object O_1_ by C-1, in a certain range of displacement deviations occurring in the transversal and longitudinal directions, C-2 also works well in creating an optical illusion. In addition, we also find, no matter object O_1_ is transferred into O_2_ or not by C-2, the cross mark placed between object O_1_ and CCD is always visible (dashed-line circled cross at the top left corner of each picture), meaning that C-2 also just plays the role of transferring object O_1_ into O_2_ but has no effect on other objects nearby O_1_.

All experimental results (Figs. 3–5) agree well with the numerical simulations based on computer generated holography^35^ (Supplementary Figs. 1–3), indicating that both numerical and experimental results verify that our approach to hiding objects and creating illusions above a carpet-like device is feasible.

## Discussions

Recently, unidirectional invisibility devices based upon carpet cloaks^36^ and parity-time symmetric structures^37^,^38^,^39^,^40^,^41^ are also inspiring some specific researching interests due to their relatively simplifying design and specific application potentials in wide fields such as directional military detection and biomedical imaging etc. For simplification, we only experimentally fabricate carpet-like devices that just work in one direction. However, our carpet-like devices can, in principle, be easily expanded to work at different directions by using multiple reference beams from different directions to construct the carpet-like devices, as we did in the cases of transmission observation^42^. In addition, by using panchromatic holographic plates to construct the carpet-like devices, we can also make them working at multiple frequencies.

In conclusion, we have reported a kind of invisibility carpet-like devices to conceal objects and create illusions above them. The devices are experimentally realized by recording the reflection holograms of objects. When illuminated by a conjugated beam to the reference light in the recording procedure, the carpet-like devices can produce a time-reversed signal to conceal objects or further to create an additional light field so as to make one object looking as another one. The carpet-like devices can work for macroscopic objects (at centimeter scale) at visible wavelength when the distance between the objects and the carpet-like devices is at the decimeter scale. In addition, the devices are made of commercially available all-dielectric materials. We believe that our work may open up a practical door to realizing invisibility and creating illusions and may inspire interesting applications in the fields such as magic camouflaging and anti-detection etc.

## Methods

Objects O_1_ and O_2_ are both made of transmission holographic plates^31^. To fabricate O_1_, we mask a transparent film with two opaque heart-patterns printed on it on a holographic plate when exposing the plate by He-Ne laser with wavelength λ = 632.8 nm. After exposing, the plate is developed by D-19, bleached by mercuric chloride bleaching liquid and illuminated by a mercury vapor lamp in the dark room to change the refractive index profile of photosensitive materials on the plate. After fixed by F-5 and bleached by R-10, the plate is fabricated to be the transparent object O_1_. (The formulas are detailed in Ref. 31.) To fabricate O_2_, a black film with a transparent smiling-face picture is masked on a holographic plate in the exposure process. Because O_2_ is an amplitude-modulated object, the two bleaching steps are not necessary in the post processing procedures, but all the other procedures maintain the same as those for creating O_1_.

The power of He-Ne laser is 60mW before beam expanding. The carpet-like devices are moved by a step motor with 2.5 μm/step and rotated by a step motor with 0.1°/step.

## Author Contributions

Q.L.C. performed the experiments, K.D.W. performed the theoretical analysis, Y.L.S. assisted the experiments, H.W. and G.P.W. designed and conducted the experiments, G.P.W. conceived the idea and supervised the project. All authors contributed to the final version of the manuscript.

## Supplementary Material

Supplementary Information Supplementary Information

Supplementary Information Movie 1

Supplementary Information Movie 2

## Figures and Tables

**Figure 1:**
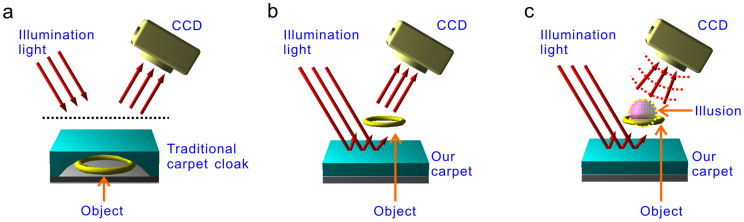
Working models of traditional carpet cloaks and our carpet-like devices. (a) A traditional carpet cloak is used to hide an object beneath. The light reflected by the under surface of the carpet cloak is distorted by the carpet layer so that the curved surface is detected as a plane mirror to the outside. Thus, objects placed in the cavity covered by the curved surface are invisible through reflection observation. (b) and (c), The present carpet-like devices are used to hide objects above them and create an illusion of transferring one object into another one. When illuminated by a light beam, the devices produces a time-reversed signal to compensate for the scattering effect of objects placed above the devices in the case of hiding the objects (b), and to further produce additional light field of another object simultaneously so as to make one object looking as another one by reflection observation (c).
