# Supplementary material for: Directionally hiding objects and creating illusions above a carpet-like device by reflection holography
Source: Sci Rep. 2015 Feb 26;5:8581. doi: 10.1038/srep08581 (PMC4341210; doi:10.1038/srep08581)
Supplement: Supplementary Information [file srep08581-s1.pdf]

SUPPLEMENTARY INFORMATION

Directionally hiding objects and creating illusions above a carpet-like device by reflection holography

Authors: Qiluan Cheng[1], Kedi Wu[1], Yile Shi[3], Hui Wang[3], and Guo Ping Wang[1, 2*]

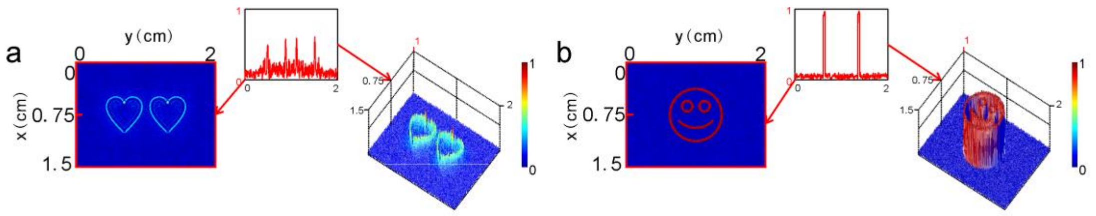

Supplementary Figure 1: Numerical simulations of objects. a-b, Planar images of objects O_1 and O_2 (left hand panels) and their 3D intensity profiles (right hand panels). Insets of each picture are the line graphs of the corresponding images along the *y* axis at *x*=0.75cm. The four peaks in the inset of **a** correspond to the edges of the two hearts, while the two peaks in the inset of **b** are the two edges of the smiling face.

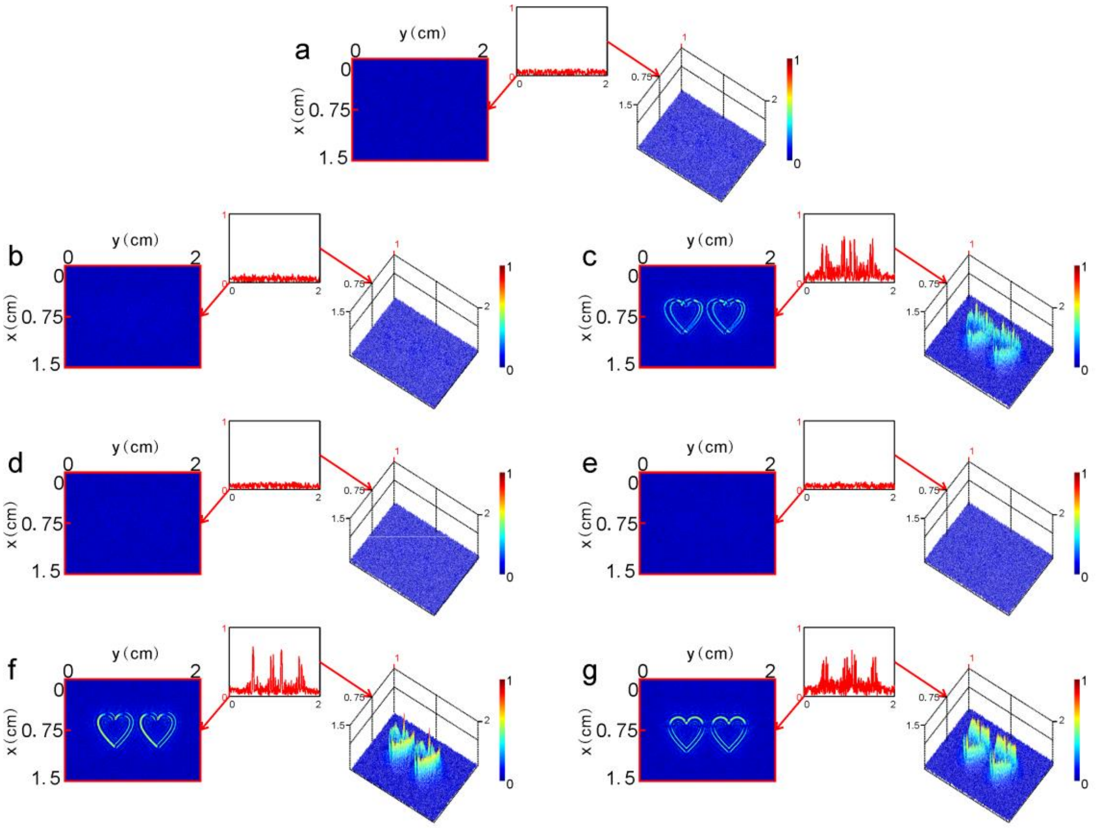

Supplementary Figure. 2. Numerical simulations of the characterization of C-1. The same as Fig. 4. A signal-noise ratio of 10:1 is introduced in the simulations.

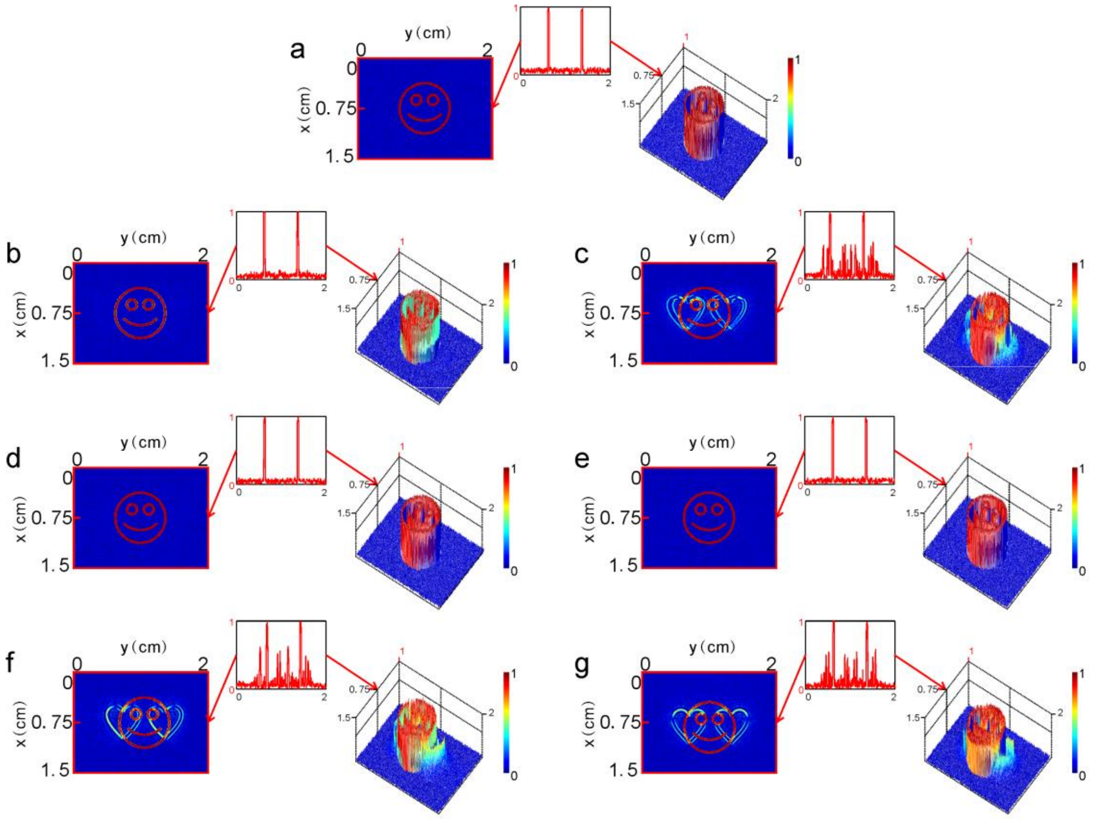

Supplementary Figure. 3. Numerical simulations of the characterization of C-2. The same as Fig. 5. A signal-noise ratio of 10:1 is introduced in the simulations.

D-19	Distilled water	Metol	Anhydrous sodium sulfite	Hydroquinone	Anhydrous sodium carbonate	Potassium bromide	Add distilled water to
	800 ml	2 g	90 g	8 g	48 g	5 g	1000 ml

bleaching liquid	Distilled water	cupric bromide	potassium persulfate	citric acid	potassium bromide	Add distilled water to
	800 ml	1 g	4 g	50g	20g	1000 ml

Supplementary Table 1: Formulas for D19 (developer), and bleaching liquid. All the chemicals listed in the table are analytically pure.

Operation	Time and temperature
Developed in D-19 (in the darkroom)	<3 min (20°C)
Rinsed in running water (in the darkroom)	3 min (18°C-20°C)
Bleached in bleaching liquid (in the darkroom)	3 min (18°C-20°C)
Rinsed in running water	10 min (18°C-20°C)

Supplementary Table 2: Postprocessing procedures. The finished holograms are air dried in room temperature.

Movie legends:

Supplementary Movie 1

This movie illustrates the dynamic process of hiding object O_1 by C-1 when C-1 is illuminated by plane light with 632.8nm on the front surface. The object is first directly illuminated by reflected plane light and then concealed by C-1 when C-1 is inserted in plane P0 properly (QuickTime; 725 KB).

Supplementary Movie 2

This movie shows the dynamic process of creating the illusion of transferring object O_1 into object O_2 by C-2 when C-2 is illuminated by plane light with 632.8nm on the front surface. The object O_1 is first directly illuminated by plane light and then transformed into object O_2 by C-2 when C-2 is inserted in plane P0 properly (QuickTime; 714 KB).
